# Preventive Effects of *Eleutherococcus senticosus* Bark Extract in OVX-Induced Osteoporosis in Rats

**DOI:** 10.3390/molecules18077998

**Published:** 2013-07-08

**Authors:** Dong Wook Lim, Jae Goo Kim, Youngseok Lee, Seok Ho Cha, Yun Tai Kim

**Affiliations:** 1 Functionality Evaluation Research Group, Division of Metabolism and Functionality Research, Korea Food Research Institute, Seongnam 463-746, Korea; 2 Department of Advanced Fermentation Fusion Science & Technology, Kookmin University, Seoul 136-702, Korea; 3 Department of Biomedical Technology, College of Medicine, Inha University, Incheon 400-712, Korea

**Keywords:** *Eleutherococcus senticosus*, osteoporosis, bone, ovariectomized rat

## Abstract

*Eleutherococcus senticosus* (Siberian ginseng), has been used as a powerful tonic herb with an impressive range of health benefits. This medicinal herb has been commonly used to treat bone metabolism diseases due to its traditional Korean medicine use to strengthen muscle and bone. This study was conducted to investigate prevention of bone loss by a standardized extract of dried *E. senticosus* stem bark in an ovariectomized (OVX) rat model of osteoporosis. The OVX groups were divided into five groups treated with distilled water, 17β-estradiol (E2 10 μg/kg, once daily, i.p) and dried stem bark of *E. senticosus* extracts (DES 10, 30, and 100 mg/kg, once daily, p.o) for eight weeks, respectively. After eight weeks of treatments, the femur bone mineral density of the 100 mg/kg DES-treated group was significantly higher than that of the OVX-control group (16.7%, *p* < 0.01) without affecting the body, organs, and uterus weights, and serum estradiol levels. Additionally, bone markers such as serum ALP, CTx, and OC levels were significantly decreased in the DES 100 mg/kg treated group. These results show that DES is able to prevent OVX-induced in bone loss without the influence of hormones such as estrogen.

## 1. Introduction

Osteoporosis is characterized by reduced bone mass and alteration in bone architecture, resulting in increased fracture risk [1]. The most common type of osteoporosis is the bone loss associated with estrogen deficiency in postmenopausal women [2]. Secondary osteoporosis from medical conditions or treatments that interfere with the attainment of peak bone mass or may predispose to accelerated bone loss [3]. Hormone replacement therapy (HRT) has proven to be efficacious in preventing bone loss and reducing the incidence of skeletal fractures in postmenopausal women [4], however, long-term HRT increases the high risk of breast and endometrial cancer, thromboembolic events and vaginal bleeding [5]. Bisphosphonates are potent inhibitors of bone resorption, and there is a variety of secondary osteoporosis where they produce substantial increases in bone density and reduce fracture risk [6]. Bisphosphonates has also been reported adverse effects such as esophageal cancer, osteonecrosis of the jaw, atypical femoral fractures [7]. Concerns about these adverse effects of osteoporosis therapies have led to interest in the alternative therapies for the prevention of osteoporosis from natural sources [8,9,10,11].

*Eleutherococcus senticosus*, known as Siberian ginseng, is a medicinal herb that belongs to the family Araliaceae and known as a powerful tonic herb with an impressive range of health benefits. This medicinal herb has been commonly used to treat bone metabolism diseases due to its traditional Korean medicine use to strengthen muscle and bone and tonify *qi* [12]. *E. senticosus* has also been reported to process anti-inflammatory [13], anti-tumor [14], anti-depressive [15], anti-steatosis [16], and neuro-protective [17] effects. The major active compounds of *E. senticosus* responsible for its diverse biological activities are eleutheroside E, chiisanoside, isofraxidin, acanthosides, daucosterol and sesamin [18].

Pro-inflammatory cytokines such as interleukin-1 (IL-1), IL-6, and tumor necrosis factor-α (TNF-α) are well known regulators of bone metabolism. These cytokines are known as highly potent bone resorption cytokines [19,20,21] and may mediate increased bone turnover markers [22] From the above reports, it is hypothesized that the anti-inflammatory action of *E. senticosus* [13,17,23] might have potential anti-osteoporotic effects in an animal model via inhibition of bone turnover markers. In addition, it was reported that *E. senticosus* extracts reduced the urinary excretion of calcium and hydroxyproline in glucocorticoid-induced osteoporotic rats [24], the most common form of secondary osteoporosis [25]. Although this study suggested that *E. senticosus* has bone health effects in secondary osteoporosis, it has also been reported that after 6 month treatment with *E. senticosus* leaves extracts no significant changes in bone mineral density (BMD) were observed in Korean post-menopausal women [26]. Since currently we lack concrete evidence to support any beneficial effect of *E. senticosus* intake on osteoporosis, its efficacy needs to be scientifically evaluated using *in vivo* experiments.

In the present study we examined the prevention of bone loss a standardized extract of dried stem bark of *E. senticosus* (DES) in an OVX-induced osteoporosis rat model. To determine whether DES treatment prevents bone loss in OVX rats, we administered the DES treatment to rats in pre-osteoporosis state. Bone mineral density (BMD) of the femur was determined weekly using dual energy X-ray absorptiometry (DEXA). Serum alkaline phosphatase (ALP) concentration was measured by a biochemistry analyzer, and telopeptide of collagen type I (CTx) and osteocalcin (OC) concentrations were assayed using a rat ELISA kit. Serum estradiol concentration was also determined by a radioimmunoassay (RIA) kit.

## 2. Results and Discussion

### 2.1. HPLC Chromatograms for Standardization of DES

**Figure 1:**
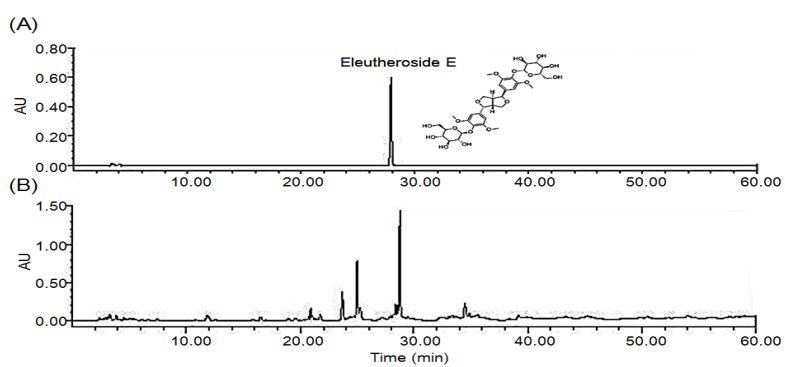
2-D HPLC chromatograms of eleutheroside E (A) and DES (B). Detection was performed by using a photodiode array detector at 205 nm. X-axis is retention time (min); Y-axis is absorbance unit (AU).

Dried stem bark of *E. senticosus* extracts (DES) was monitored at 205 nm for eleutheroside E (Figure 1). The content of eleutheroside E was calculated for standardization. DES was standardized to contain 0.48 ± 0.05% eleutheroside E.

### 2.2. Weekly Body Weight in Treatments of DES

**Figure 2:**
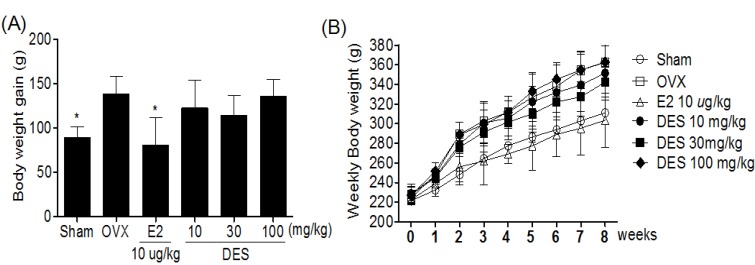
Effects of DES on body weight (**A**) and body weight gain (**B**) in OVX rats. The body weight was recorded weekly during the experimental period. The body weight gain was calculated by the equation: final body weight - initial body weight. Data are mean ± SD values (n = 10 per group). * *p* < 0.05, significantly difference from the OVX-control group.

Body weights increased over time in all groups, but body weights increased significantly more in the OVX alone groups than in sham groups. A significant difference in body weight was observed between the E2 10 μg/kg treated group and the OVX-control group by three weeks after initiating administration (Figure 2B). After eight weeks of treatments, the body weight gain of the E2 10 μg/kg treated group was also significantly less than that of the OVX-control group. However, there was no significant difference in the body weight and body weight gain of DES-treated groups during the experimental period (Figure 2A).

### 2.3. Bone Mineral Density of the femur in Treatments of DES

**Figure 3:**
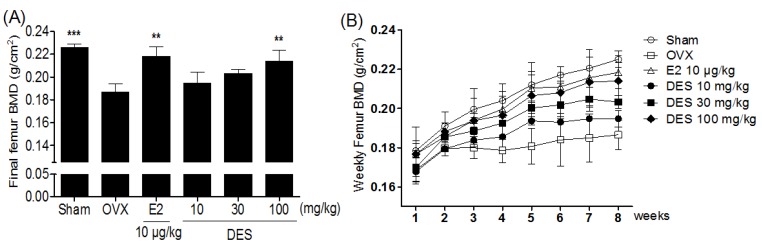
Effects of DES on change of final femur BMD (**A**) and weekly femur BMD (**B**) in OVX rats. These BMD values were determined weekly during the experimental period by dual energy X-ray absorptiometry. Data are mean ± SD values (n = 10 per group). ** *p* < 0.01, *** *p* < 0.00, significantly difference from the OVX-control group.

Three weeks after the OVX operation, OVX-control group showed a significant decrease in the femur bone mineral density (BMD) compared to the sham group (*p* < 0.05) (Figure 3B). After eight weeks of treatments, the final femur BMD of the 100 mg/kg DES-treated group was significantly higher than that of the OVX-control group (16.7%, *p* < 0.01, Figure 3A).

### 2.4. Uterus and Organ Index in Treatments of DES

**Figure 4:**
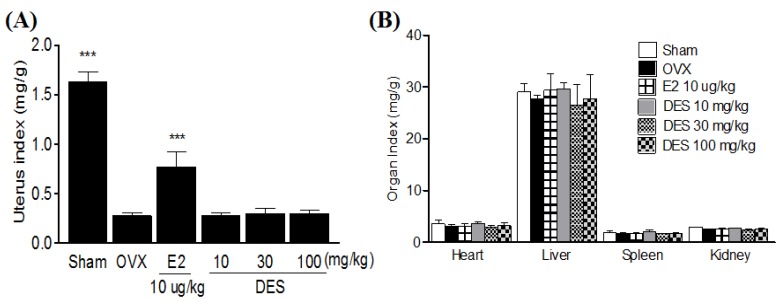
Effects of DES on change in uterus (**A**) and organ index (**B**) in OVX rats. Uterus and organs were dissected, washed with saline, and immediately weight for analysis. Data are mean ± SD values (n = 10 per group). *** *p* < 0.001, significantly difference from the OVX-control group.

OVX caused atrophy of uterine tissue, indicating the success of the surgical procedure and in the E2 10 μg/kg treated group the uterus index (mg/g) increased significantly compared to the OVX-control group. However, DES-treated groups did not show an effect on the uterus index following OVX (Figure 4A). The heart, liver, spleen, and kidney indexes were not significantly different in each group either (Figure 4B).

### 2.5. Serum Bone Marker in Treatments of DES

**Figure 5:**
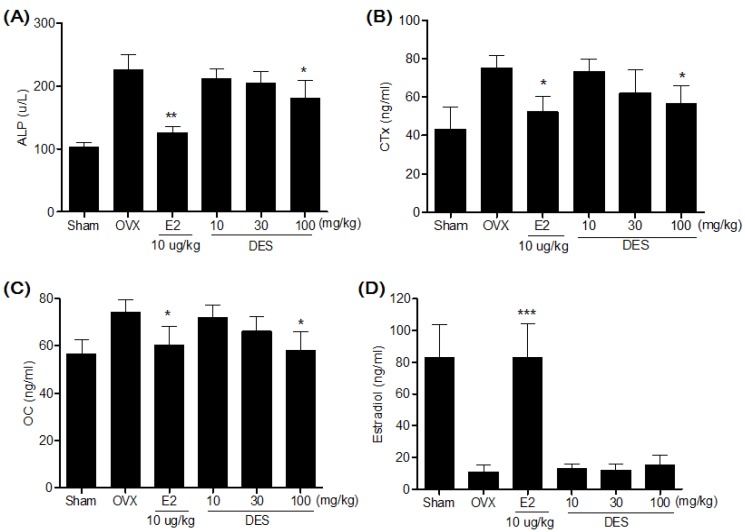
Effects of DES on serum ALP (**A**), CTx (**B**), OC (**C**) and estradiol (**D**) concentrations. Data are mean ± SD values (n = 10 per group). * *p* < 0.05, ** *p* < 0.01, *** *p* < 0.001, significantly difference from the OVX-control group.

Serum ALP, CTx and OC concentrations in the OVX-control group were significantly higher compared to the sham group. After eight weeks treatments, the DES 100 mg/kg treated group showed significantly lower serum ALP, CTx and OC concentrations compared to the OVX-control group (Figure 5). In case of serum estradiol, the DES treated groups were not significantly different from the OVX-control group (Figure 5D).

### 2.6. Discussion

Our finding demonstrated that eight weeks of treatment with DES significantly decreased the BMD loss in the femur and inhibited the bone markers such as serum ALP, CTx, and OC levels compared to the OVX-control group without the influence of hormones such as estrogen. Bone loss caused by estrogen deficiency in both humans and experimental animals is primarily due to an increase in osteoclastic bone resorption [27]. OVX rats, which exhibit most of the characteristics of human postmenopausal osteoporosis [28] have been widely used as a model for the evaluation of potential osteoporosis treatments [29].

Like previous reports, our OVX resulted in significantly decrease in the femur BMD after eight weeks. This BMD loss was accompanied by a significant increase in bone remodeling, as evidenced by the enhanced biochemical bone turnover markers. These markers have been widely used as a research tool to measure the effects of various drugs on bone remodeling [30]. A correlation between the serum levels of OC [31] and ALP [32], two sensitive markers of bone formation, and CTx [32], a measure of bone resorption, with microarchitecture has been reported in studies of postmenopausal women and laboratory animals [33,34]. In the present study, oral administration of DES at dosages of 100 mg/kg significantly decreased the BMD loss in the femur, which was reflected by the decrease in serum ALP, CTx, and OC levels compared to the OVX-control group. These results suggest that DES decreases bone loss by inhibiting bone remodeling in OVX rats.

OVX dramatically increases body weights, while E2 treatment presents completely normal levels [35]. Although the mechanisms by which OVX induces an increase in body weight are not clear, estrogen deficiency induced body fat accumulation and subsequently caused an increase in body weight [36]. Heine *et al*. demonstrated that estrogen receptor (ER) knockout mice have higher fat mass and lower energy expenditure than wild-type mice [37]. Estrogen may be involved directly in rat energy metabolism by binding to ER within the abdominal and subcutaneous fat tissues [38]. Estrogen expresses its activities by binding to different ERs, ERα and ERβ. ERβ is more abundant than ERα in bone tissue while ERα is mainly distributed in reproductive cells and is the dominant receptor mediating the most obvious effects of E2 in breast and uterus [39]. In our results, oral administration of DES did not affect body weight gain, uterotrophic activity, and serum estradiol concentration in OVX rats. Consistent with our finding from the E2 treated group, the DES might have anti-osteoporotic effects in OVX rats, without the influence of hormones such as estrogen. However, further mechanistic studies are needed to clarify whether the prevention of bone loss effects of DES may be elicited by regulating the expressions of ERβ.

## 3. Experimental

### 3.1. Sample Preparation and HPLC Analysis

Dried stem bark of *E. senticosus* was purchased from the Kapdang Co. (Seoul, Korea). The sample was identified by Dr. Seok Ho Cha and a voucher specimen (#NP-1033) was deposited in the Department of Biomedical Technology, College of Medicine, Inha University, Incheon, Korea. The dried stem bark of *E. senticosus* (300 g) was extracted with 70% ethanol (3,000 mL) for 3 h at 80 °C in a reflux apparatus. The extracts were filtered and concentrated under reduced pressure, and samples were lyophilized to yield a dark yellow powder. The yield of dried stem bark of *E. senticosus* extract (DES) was 10.5%. The standardization of DES was performed by a high performance liquid chromatography (HPLC) system equipped with a Waters 1525 pump, a 2707 auto sampler and a 2998 photodiode array detector (PDA) detector. The separation was achieved at 30 °C on Waters Sunfire™ C18 (250 mm × 4 mm i.d., 5 μm particle size) column. DES was monitored at 205 nm for eleutheroside E. The run time was set at 60 min and flow rate was 1.0 mL/min (sample injection volume, 10 μL). The mobile phase A and B were 1% phosphoric acid (H_3_PO_4_) (v/v) and acetonitrile (CH_3_CN), respectively. The gradient elution profile was as follows: 0–25 min 5–50% B; 25–40 min 50–70% B; 40–60 min 70% B.

### 3.2. Animals and Treatments

Female Sprague-Dawley (SD) rats, 8-weeks old, were purchased from Samtako (Gyeonggi-do, Korea). Animals were housed at two rats per cage in an air-conditioned room at 23 ± 1 °C, 55–60% relative humidity, and a 12 h light/dark cycle (07:00 lights on, 19:00 lights off), and were given a laboratory regular rodent diet. All animal experiments were carried out according to the guidelines of the Korea Food Research Institutional Animal Care and Use Committee (KFR-M-13003). After acclimatization for 1 week, 9-week-old female SD rats were anesthetized with 2% of isoflurane and ovaries were removed bilaterally. A sham operation, during which the ovaries were just touched with forceps, was performed on the sham group. A recovery period of 1 week after surgery, rats were divided into six following treatment groups: (1) sham + vehicle, (2) OVX + vehicle, (3) OVX + 17*β*-estradiol (E2, 10 μg/kg once daily, i.p), (4) OVX + DES 10 mg/kg, (5) OVX + DES 30 mg/kg, (6) OVX + DES 100 mg/kg. The dosages of DES were selected in consideration of usual human dosages (40–545 mg/60 kg-weighted human, extracts dosages) for alternatives to traditional hormone replacement in postmenopausal women [40]. DES at a dosage of 10 mg/kg in rats corresponds to 0.6 g DES/60 kg-weighed human subject, where DES extracted from approximately 5.8 g of the DES raw material; this concentration is similar to dosage of *E. senticosus* and *Panax ginseng* (equivalent to 4 g and 2 g/day of raw material; dried root, respectively) in drinking supplementation for clinical trials (Mediherb Pty. Ltd. Warwick Queensland, Australia) [41]. Finally, we decided the dosages of DES, *i.e.*, 10, 30, and 100 mg/kg, separated by three time intervals. 

DES was dissolved in distilled water for oral administration at the desired doses in a volume of 2 mL/kg once daily. E2 dissolved in distilled water, with 1% DMSO and 0.1% Tween 20. All groups were treated for eight weeks. During the experimental period, body weight and femur BMD were determined weekly. At the end of the treatment period, the rats were fasted for 12 h, and blood was collected via the abdominal aorta. Uterus tissue and other organs were dissected, washed with saline solution, and weighted for analysis. Uterus and organ indexes (mg/g) were calculated by dividing the uterus and organ weights by the body weight.

### 3.3. Bone Mineral Density Measurements

The BMD of rat femurs was measured by a PIXImus (GE Lunar PIXImus, GE Healthcare, Madison, WI, USA), dual energy X-ray absorptiometer (DEXA), equipped with appropriate software for bone density assessment in small laboratory animals. Calibration of the instrument was conducted as recommended by the manufacturer. Quality control with BMD (0.0553 g/cm^2^) and percentage fat composition (16.7%) of the phantom were also performed each time the instrument was switched on. All rats were placed in the same direction.

### 3.4. Serum Estradiol and Bone marker Analysis

The serum samples were prepared by centrifugation of the collected blood samples (1,013 g for 15 min at 4 °C), then stored at −80 °C for biochemical determinations. Serum ALP concentrations were measured by VetTest 8008 (IDEXX Lab Inc., Westbrook, ME, USA). Serum hormone level was determined by radioimmunoassay (RIA). The estradiol RIA was performed according to the instructions accompanying a Coat-a-Count kit (Diagnostic Products, Los Angeles, CA, USA). Serum concentrations of the bone turnover marker osteocalcin (OC) were assayed using a rat ELISA kit (Metra OC, Quidel Corporation, San Diego, CA, USA). Serum levels of telopeptides of collagen type I (CTx), that correlate with bone resorption, with high levels indicating excessive osteoclastic activity were analyzed using commercial ELISA kits (Serum CrossLaps, Nordic Bioscience, Herlev, Denmark; Metra Serum Pyd, Quidel Corporation).

### 3.5. Statistical Analysis

All data were presented as the mean ± standard deviation (SD). The effects of different treatments were compared by one-way ANOVA test, followed by the post-hoc Tukeytest for multiple comparisons using GraphPad Prism 5 (GraphPad Software Inc., La Jolla, CA, USA). *p* < 0.05 was considered statistically significant.

## 4. Conclusions

In conclusion, DES is able to prevent OVX-induced in bone loss without the influence of hormones such as estrogen, suggesting that DES may be a reasonable natural alternative for the prevention of postmenopausal osteoporosis.
